# High Bone Mass is associated with bone-forming features of osteoarthritis in non-weight bearing joints independent of body mass index

**DOI:** 10.1016/j.bone.2017.01.005

**Published:** 2017-04

**Authors:** C.L. Gregson, S.A. Hardcastle, A. Murphy, B. Faber, W.D. Fraser, M. Williams, G. Davey Smith, J.H. Tobias

**Affiliations:** a Musculoskeletal Research Unit, School of Clinical Sciences, University of Bristol, UK; b MRC Integrative Epidemiology Unit, University of Bristol, Bristol, UK; c Department of Medicine, Norwich Medical School, University of East Anglia, Norwich, UK; d Department of Radiology, North Bristol NHS Trust, Bristol, UK

**Keywords:** Osteoarthritis, Osteophyte, Bone, Hand, Epidemiology, X-ray

## Abstract

**Objectives:**

High Bone Mass (HBM) is associated with (a) radiographic knee osteoarthritis (OA), partly mediated by increased BMI, and (b) pelvic enthesophytes and hip osteophytes, suggestive of a bone-forming phenotype. We aimed to establish whether HBM is associated with radiographic features of OA in non-weight-bearing (hand) joints, and whether such OA demonstrates a bone-forming phenotype.

**Methods:**

HBM cases (BMD Z-scores ≥  + 3.2) were compared with family controls. A blinded assessor graded all PA hand radiographs for: osteophytes (0–3), joint space narrowing (JSN) (0–3), subchondral sclerosis (0–1), at the index Distal Interphalangeal Joint (DIPJ) and 1st Carpometacarpal Joint (CMCJ), using an established atlas. Analyses used a random effects logistic regression model, adjusting *a priori* for age and gender. Mediating roles of BMI and bone turnover markers (BTMs) were explored by further adjustment.

**Results:**

314 HBM cases (mean age 61.1 years, 74% female) and 183 controls (54.3 years, 46% female) were included. Osteophytes (grade ≥ 1) were more common in HBM (DIPJ: 67% *vs.* 45%, CMCJ: 69% *vs.* 50%), with adjusted OR [95% CI] 1.82 [1.11, 2.97], p = 0.017 and 1.89 [1.19, 3.01], p = 0.007 respectively; no differences were seen in JSN. Further adjustment for BMI failed to attenuate ORs for osteophytes in HBM cases *vs.* controls; DIPJ 1.72 [1.05, 2.83], p = 0.032, CMCJ 1.76 [1.00, 3.06], p = 0.049. Adjustment for BTMs (concentrations lower amongst HBM cases) did not attenuate ORs.

**Conclusions:**

HBM is positively associated with OA in non-weight-bearing joints, independent of BMI. HBM-associated OA is characterised by osteophytes, consistent with a bone-forming phenotype, rather than JSN reflecting cartilage loss. Systemic factors (*e.g.* genetic architecture) which govern HBM may also increase bone-forming OA risk.

## Highlights

• High Bone Mass (HBM) is characterised by osteophytosis in non-weight-bearing joints of the hand.
• Hand osteophytosis is not explained by the higher BMI or by the lower bone turnover seen in HBM.
• HBM-associated OA, characterised by osteophytes, is consistent with a ‘bone forming’ phenotype.
• A similar ‘bone forming’ phenotype has been observed in weight-bearing joints of the knee and hip in HBM.
• Systemic factors, *e.g.* genetic architecture, which govern HBM may also increase the risk of osteophytosis.

## 1 Introduction

Epidemiological studies have consistently demonstrated an association between higher bone mineral density (BMD) and both prevalent [1], [2], [3], [4] and incident [5], [6], [7] radiographic osteoarthritis (OA) of large joints. To gain insights into mechanisms underlying this BMD-OA relationship, we recently studied the prevalence and phenotype of OA in a unique High Bone Mass (HBM) population. We have found that HBM individuals have a higher prevalence of self-reported joint replacement and use of non-steroidal anti-inflammatory drugs (NSAIDs) compared with family controls [8]. An increased prevalence of radiographic hip OA was also observed in HBM individuals, particularly with respect to bony features such as osteophytosis and subchondral sclerosis, whereas little evidence was seen for features reflecting cartilage loss such as joint space narrowing (JSN) [9]. Further characterisation of knee OA revealed a similar, osteophyte predominant, radiographic OA phenotype [10]. Additional evidence of a ‘bone-forming’ tendency is supported by a greater prevalence and severity of radiographic pelvic enthesophytes (bony spurs at tendon/ligament insertions) seen in HBM individuals, compared with family controls [11].

Interestingly, HBM individuals also tend to have a greater body mass index (BMI) [12], and women with HBM have elevated fat mass on total body DXA with a particular tendency towards central adiposity [13]. Whilst genetic sequencing is ongoing, HBM cases are thought to have a genetic predisposition to their raised BMD suggesting a causal pathway whereby raised BMD leads to increased fat mass [13], [14]. Mouse models have suggested bone turnover directly influences insulin sensitivity and adiposity *via* a relay involving osteocalcin (an osteoblast-specific protein) and adiponectin (an adipokine produced by white adipose tissue). Reduced bone turnover, resulting in decreased osteocalcin, has been associated with lower adiponectin, impaired insulin sensitivity, and increased fat deposition due to reduced energy expenditure [6]. Obesity is an established risk factor for OA, particularly in large weight-bearing joints; increased mechanical load is considered deleterious to joints [12], [15]. However, although greater BMI contributed in part to the association we observed between HBM and knee OA, the association persisted after BMI adjustment [10], and the relationship between HBM and hip OA appeared independent of BMI [9].

The observation that obesity is also associated with OA in non-weight-bearing joints, *i.e.* the hand [16], suggests adiposity may also modify OA risk *via* metabolic pathways, *e.g.* through increased circulating adipokines or chronic inflammation [17], [18]. The relative importance of these two pathways (mechanical *vs.* metabolic) in mediating the BMI-OA association remains to be determined. We aimed to establish whether HBM is associated with OA in non-weight-bearing joints, and if so whether such OA also demonstrates a bone-forming phenotype and whether increased BMI contributes to any identified association. We hypothesized that, in line with our previous findings and evidence from general population studies [11], HBM would be associated with a ‘bone-forming’ phenotype of OA in non-weight-bearing joints potentially reflecting underlying systemic factors.

## 2 Methods

### 2.1 Participant recruitment

HBM cases were recruited as part of the UK-based HBM study, a multi-centre observational study of adults with unexplained HBM; full details of DXA database screening and recruitment have previously been reported [12]. Potential index cases were initially identified by screening 13 National Health Service (NHS) DXA databases (335,115 DXA scans in total) for T and/or Z-scores ≥  + 4 at any site within the lumbar spine or hip. Previous case studies of HBM used Z-score thresholds to define HBM [19]; however, as Hologic DXA scanner databases store T- but not Z-scores, our search was of T- and/or Z-score ≥ + 4. All DXA images were inspected by trained clinicians to exclude scans with artefactual elevation of DXA BMD, resulting in 49.4% of scans being excluded due to degenerative disease/osteoarthritis/scoliosis, and a further 15.5% for other reasons including surgical/malignant/Pagetic artefacts *etc.* To reduce contamination of our remaining DXA scans by more moderate OA, we aimed to refine our case definition based upon restriction to specific lumbar verterba(e). At our largest centre 463 antero-posterior DXA scans with T/Z-score ≥ + 4 were graded for OA severity by lateral Kellgren & Lawrence scores, and examined in relation to BMD at lumbar vertebral levels [20], [21]. Total L1-L4 Z-score was strongly associated with increasing KL score (β coefficient 1.01 [0.54, 1.48], p < 0.001) [21]; in contrast to other lumbar vertebrae, L1 Z-score was not associated with the presence of OA (0.04 [− 0.53, 0.60], p = 0.89) [21], reflecting the recognized pattern of progressive OA changes seen in descending sequential lumbar vertebrae [22], [23]. Nor did total hip Z-score reflect lumbar spine OA [21]. As a generalised HBM trait is expected to affect both spine and hip BMD, though not necessary to the same extent, we refined our HBM index case definition as having either a) L1 Z-score ≥ + 3.2 plus total hip Z-score ≥ + 1.2 or b) total hip Z-score ≥ + 3.2 plus L1 Z-score ≥ + 1.2. A + 3.2 threshold was consistent with the only published precedent for identifying HBM using DXA [24] and most appropriately differentiated generalised HBM from artefact. Z rather than T-score limited age bias.

Further HBM cases were identified through DXA assessment of the relatives and spouses of index cases. In first-degree relatives, HBM was defined as a summed L1 Z-score plus total hip Z-score ≥ + 3.2 (12). BMD was standardized using established formulae [25], [26]. 41% of relatives screened were affected and combined with HBM index cases, with remaining unaffected first-degree relatives/spouses forming a family control group. Standardized clinical assessments, performed by a doctor or research nurse, including a structured interview, clinical examination and weight and height measurement, were identical in both HBM cases and controls. Concurrently, PA dominant hand X-rays were performed in all participants according to local protocols at each centre. Those reporting joint pain/aching/stiffness [27] for months/years in the X-rayed hand were defined as having a clinical history consistent with OA. Recruitment ran July 2005–April 2010. Written informed consent was obtained from all participants in line with the Declaration of Helsinki [28]. Participants were excluded if aged < 18, pregnant or unable to provide written informed consent for any reason. This study was approved by the Bath Multi-centre Research Ethics Committee (REC reference 05/Q2001/78) and at each NHS Local REC.

### 2.2 Assessment of radiographs

All available HBM case and control radiographs, collected across all study centres, were pooled for assessment. Files were relabelled and presented in a random order to ensure blinding of the assessor. Radiographs were graded by a single observer (AM) following training by an individual with previous experience of radiographic OA scoring (SH). X-ray images (all PA) were stored as DICOM files and viewed using open source ImageJ software [29]; semi-quantitative assessments were recorded.

We assessed two joints in the hand; the index Distal Interphalangeal Joint (DIPJ) and 1st Carpometacarpal Joint (CMCJ), chosen as they are the most commonly affected by OA (in women, and our population is predominantly female) [30]. Each joint was assigned a semi-quantitative grading of individual radiographic features of OA (osteophytes, joint space narrowing and subchondral sclerosis) using the established Altman atlas [31], and the presence or absence of mal-alignment (at the DIPJ), and cysts (at the CMCJ) (Table 1). Categorical scores for the individual radiographic features were converted to binary variables for analysis.

Image quality was rated by the operator at the time of assessment (good, poor, very poor); very poor X-rays, judged in terms of penetration or resolution, were excluded (n = 3). At the end of the study 40 randomly selected hand X-rays were re-graded by the primary observer (AM) and by a secondary observer (SH), to assess intra-rater and inter-rater repeatability respectively. Unweighted intra-rater kappa values for the above listed binary variables were all ≥ 0.60 (considered substantial agreement [32]), except CMCJ JSN where binarized as ‘moderate’ (JSN grade ≥ 2) (0.53 [95% CI 0.07, 1.00]), but when binarized as ‘any JSN’ (JSN grade ≥ 1) agreement was better (0.74 [95% CI 0.53, 0.95]); DIPJ mal-alignment and CMCJ cysts were not seen amongst this subgroup. The inter-rater kappas for all measurements were > 0.60, except CMCJ subchondral sclerosis which was rarely seen (0.23 [95% CI − 0.21, 0.67]).

### 2.3 Assessment of covariates

Values for age (at time of X-ray), gender and BMI were obtained. BMI was calculated as weight (kg)/height (metres^2^). Two non-fasted EDTA samples were collected and plasma separated and frozen within 4 h to − 80 °C. Bone formation (Procollagen type 1 amino-terminal propeptide [P1NP], total osteocalcin) and resorption (β-*C*-telopeptides of type I collagen [βCTX]) markers were measured. All had inter- and intra-assay coefficients of variation < 6.0% across the assay working ranges. Electrochemiluminescence immunoassays (ECLIA) (Roche Diagnostics, Burgess Hill, UK) were used to measure plasma concentrations of P1NP, osteocalcin, and βCTX (detection limits 4.0, 0.6, 0.01 μg/L respectively).

Current and life-time physical activity (PA) was measured by postal questionnaire comprising (i) the short last 7 days International PA Questionnaire (IPAQ2002, http://www.ipaq.ki.se/ipaq.htm [33], [34]) and (ii) historical PA questionnaire [35], [36], [37]. 87.3% completed PA questionnaires: those who did not respond had similar anthropometric characteristics to those who did (Supplementary Table 1).

### 2.4 Statistical analysis

Demographic statistics for the HBM cases and family controls were summarised as mean (SD) for continuous variables and counts (percentages) for categorical variables. Categorical variables were initially cross-tabulated and percentages calculated: the chi-squared (χ^2^) test was used to assess the association between binary variables, and the unpaired *t*-test to compare mean values (or normally distributed variables) between cases and controls. Associations between HBM case status and binary radiographic OA outcomes were analysed using logistic regression in a random effects model, to allow for the lack of statistical independence due to within-family clustering of environmental factors and shared genotypes. Analyses were adjusted for the *a priori* confounders; age and gender, then additionally for (i) BMI and (ii) bone turnover, as potential mediators. The potential influence of other potential confounders was assessed. Odds ratios before and after adjustment are presented with 95% confidence intervals (95% CI). Analyses were repeated stratified by gender.

Pre-planned sensitivity analyses comprised: i) exclusion of poorer quality X-rays, ii) excluding HBM cases/controls with self-reported inflammatory arthritis, iii) excluding HBM cases/controls with self-reported steroid use (current or historical), and iv) restricting analyses to HBM cases meeting the index case definition at the hip. Data were analysed using Stata release 12 statistical software (StataCorp, College Station, TX, USA).

## 3 Results

### 3.1 Participant selection and characteristics

**Fig. 1:**
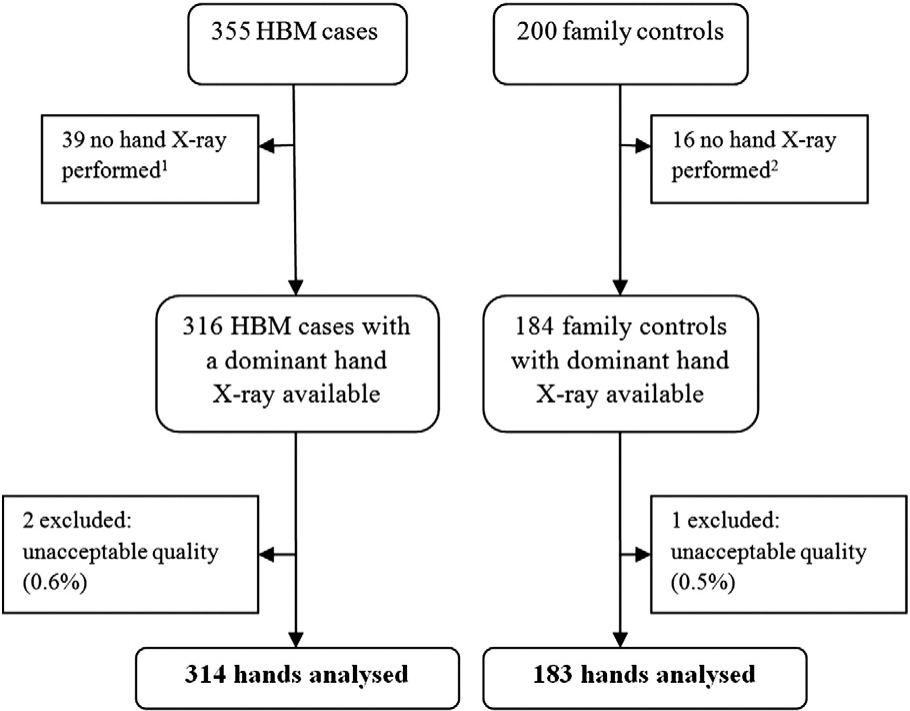
Flow diagram summarising selection of radiographs for inclusion in the study. Selection of High Bone Mass (HBM) case and family control X-rays (process of recruitment to study described previously). ^1^Reason recorded for missing X-rays in HBM cases: unable to travel (n = 7), no X-rays at study centre (n = 10), unable to attend/wait/comply (n = 3), patient declined (n = 6), reside abroad (n = 2), reason unknown (n = 11). ^2^Reason recorded for missing X-ray in family controls: unable to travel (n = 1), did not continue in study (n = 1), no X-rays at study centre (n = 4), unable to attend/wait/comply (n = 3), patient declined (n = 2), reason unknown (n = 5).

Fig. 1 summarises the selection of radiographs for inclusion in our analysis. Of the original study population 500 had X-rays performed; those who did not (n = 55) were similar in all characteristics other than they were more likely to be male, and hence were marginally taller and reported higher levels of current physical activity (Supplementary Table 2). Excluding 3 very poor quality X-rays permitted analyses in 314 HBM cases and 183 controls; demographics are detailed in Table 2. HBM cases were generally older and were more likely to be female, post-menopausal and have used estrogen replacement, and had greater BMI compared with controls. As expected, cases had substantially higher BMD and BMD Z-scores than controls (Supplementary Fig. 1). 92.8% of the study population were right hand dominant.

### 3.2 HBM and clinical hand OA

HBM cases more frequently reported a clinical history, and had examination findings, consistent with overall hand OA (Table 3); however, these findings were fully attenuated by age and gender adjustment (Table 4).

### 3.3 HBM and radiographic features of hand OA: unadjusted analyses

Osteophytes were more commonly seen amongst HBM cases than controls, at both the DIPJ and CMCJ, whether graded as any osteophyte (≥ grade 1) (DIPJ: 67% *vs.* 45%; CMCJ 69% *vs.* 50%; in HBM *vs.* controls respectively), or restricted to moderate and/or more severe osteophytes (≥ grade 2) (DIPJ: 29% *vs.* 14%; CMCJ 30% *vs.* 15%; in HBM *vs.* controls respectively), p < 0.001 for all (Table 3). No differences between groups were seen in the frequency of either any (≥ grade 1) or moderate (≥ grade 2) JSN at the DIPJ. At the CMCJ, JSN (≥ grade 1) was observed equally frequently amongst HBM cases and controls; however, more marked JSN (≥ grade 2), whilst being uncommon overall, was seen more often amongst HBM cases than family controls (11.8% *vs.* 3.8% respectively; p < 0.003). Subchondral sclerosis was uncommon overall, but observed more frequently amongst HBM cases than controls, at both joint sites (DIPJ: 8% *vs.* 3%; CMCJ 12% *vs.* 7%; in HBM *vs.* controls respectively). Although DIPJ mal-alignment and CMCJ cysts occurred more commonly amongst HBM cases, these features were rarely observed.

### 3.4 HBM and radiographic features of hand OA: analyses adjusted for age and gender

**Fig. 2:**
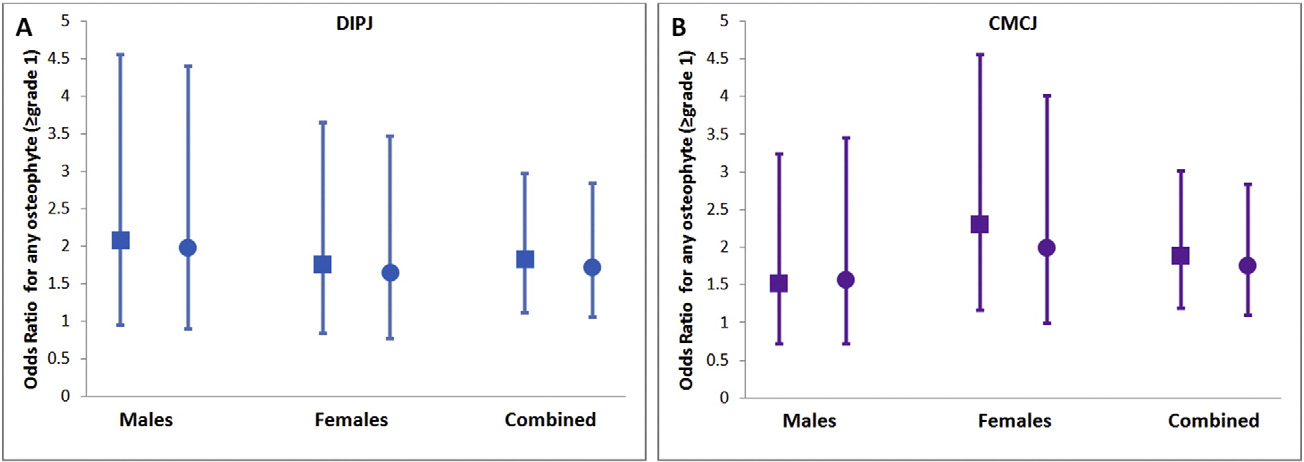
Odds ratios for osteophytes (≥ grade 1) at the index Distal Interphalangeal Joint (DIPJ) (A) and 1st Carpometacarpal Joint (CMCJ) (B); stratified by gender and combined. Square symbols represent point estimates aged for age and gender when combined. Circular symbols represent point estimates aged for age and BMI, as well as gender when combined. No evidence for a gender interaction was identified. (Males n = 179, Females n = 318, Combined n = 497.) OR and 95% CI shown.

After adjustment for age and gender clear associations persisted between HBM case status and osteophytes, with HBM cases having increased odds of ≥ grade 1 osteophytes at the DIPJ (OR 1.82 [95% CI 1.11, 2.97], p = 0.017) and CMCJ (1.89 [1.19, 3.01], p = 0.007), and of ≥ grade 2 osteophytes at the CMCJ (1.85 [1.07, 3.22], p = 0.028) (Table 4; Fig. 2). In contrast no association was seen between HBM case status and JSN, at either joint site. Furthermore, the associations between HBM case status and subchondral sclerosis, DIPJ mal-alignment and CMCJ cysts, were fully attenuated by age and gender adjustment.

### 3.5 HBM and radiographic features of hand OA: analyses with further adjustments

To determine the extent to which BMI might lie on the causal pathway between HBM and osteophytic OA, we further adjusted our age and gender model for BMI (Table 4; Fig. 2). Further BMI adjustment marginally attenuated point estimates for osteophyte associations, but otherwise similar patterns were still observed. Further adjustment for other potential confounders (menopausal status, diabetes mellitus, estrogen replacement, steroid use) widened confidence intervals but did not influence overall results (Supplementary Table 3). As physical activity was not associated with HBM status this was not considered as a potential confounder (Table 1).

To investigate the role differences in bone turnover might play in explaining the association between HBM and osteophytic OA, we analysed data from 475 individuals (96% of study population) in whom bone turnover marker measures were available, and compared our model adjusted for age, gender, BMI, with results further adjusted for CTX, P1NP and osteocalcin (Supplementary Table 4). Adjustment for bone turnover markers did not alter associations or point estimates.

### 3.6 Gender stratified analyses

Associations between HBM and radiographic features of OA at the DIPJ were similar across men and women (Supplementary Table 5). Associations between HBM and CMCJ osteophytes (both ≥ grade 1 and ≥ grade 2) appeared stronger in women than men; however, no formal evidence of a gender interaction was detected (p = 0.65 and 0.19 respectively).

### 3.7 Sensitivity analyses

25 X-rays (19 HBM cases; 6 controls) were considered to be of poor quality in terms of resolution/penetration. Excluding these from the analyses did not materially influence the observed associations (Supplementary Table 6). 22 participants reported a history of inflammatory arthritis (20 HBM cases; 2 controls). Exclusion from analyses again did not influence the observed associations (Supplementary Table 7). Exclusion of 113 individuals who reported previous and/or current use of steroids, and 12 individuals in whom we lacked data regarding steroid use, widened the confidence interval but did not materially influence the point estimate for the association between HBM status and CMCJ osteophytes ≥ grade 2, otherwise findings were unchanged (Supplementary Table 8). Amongst HBM cases, 166 individuals had a total hip Z-score < + 3.2. When comparing the remaining 148 HBM cases and 183 controls point estimates were broadly consistent with the main analysis, although confidence intervals widened. There remained a clear association between HBM status and CMCJ osteophytes ≥ grade 1 (2.03 [1.12, 3.69], p = 0.020) (Supplementary Table 9).

## 4 Discussion

We have shown an increased prevalence of radiographic features of OA in non-weight-bearing joints of the hand, amongst HBM individuals compared with family controls, similar to that we previously identified in the weight-bearing joints of the knee and hip [9], [10]. As we had hypothesized, osteophyte measures were more strongly and consistently associated with HBM, than was JSN, consistent with a ‘bone-forming’ phenotype of OA, again in line with our findings at the larger joints [9], [10]. However, unlike the association we identified between HBM and knee OA, which attenuated by approximately 50% after BMI adjustment [10], BMI adjustment did not influence HBM-osteophyte associations for any radiological grade at either the DIPJ or CMCJ. These findings suggest that HBM-osteophyte associations in the hand are independent of weight-bearing and of BMI/metabolic factors. Interestingly, despite lower bone turnover measured amongst HBM cases, further adjustment for bone turnover markers, to investigate their potential role on the causal pathway, failed to account for the HBM-osteophyte association.

Subchondral sclerosis, a further ‘bone-forming’ feature of OA, whilst uncommon overall, was more prevalent amongst HBM cases than family controls in unadjusted analyses. DIPJ mal-alignment and CMCJ cysts were also observed more frequently amongst HBM cases than controls; however, generally these were rare and fully accounted for by between-group differences in age and gender. After taking account of age, gender and BMI, overall odds ratios for osteophytes (≥ grade 1) in HBM cases *vs.* controls were similar at the DIPJ (1.72 [1.05, 2.83]), and CMCJ (1.76 [1.10, 2.83]) as previously reported at the hip (2.12 [1.61, 2.79]) [9] and the knee (1.62 [1.21, 2.15]) [10], suggesting that the increased risk of osteophytes conferred as a direct result of HBM (independent of BMI) is similar across all joint sites.

Our findings are consistent with epidemiological evidence from the general population, that increased BMD is a risk factor for hand OA [1], [38], [39]. Whilst a number of studies have assessed the relationship between BMD and radiographic OA of knees and hips, many fewer have focussed on the hand. Of those that have, most use the global score of Kellgren and Lawrence [20], rather than feature-specific grades as we have done [40]. Although, one study from 1995 examined radiographic hand OA in 300 healthy older women, deriving global osteophyte and JSN scores (graded 0–30) using 10 joints. Interestingly the osteophyte, but not JSN score was positively correlated with BMD, apparently independent of BMI although data were not shown [38]; authors speculated bone density and osteophytosis may therefore have common genetic determinants. More recently, a larger Korean study identified a positive relationship between BMD and both hand osteophytes and sclerosis, with an inverse relationship seen between BMD and JSN in the hand [41], highlighting the importance of individual grading of OA sub-phenotypes.

Extreme HBM is likely to be genetically determined with onset of elevated bone mass developing relatively early in life, likely before the onset of OA; the genetic basis of increased BMD in our HBM population is currently being investigated [42]. Potentially, the genetic influences governing BMD might also affect cartilage and OA risk. Wnt signalling is an important regulator of osteoblastic bone formation and mutations activating this pathway produce a HBM phenotype [19]. Polymorphisms in genes thought to regulate the Wnt pathway, plus reduced levels of the Wnt pathway inhibitor DKK1, have both been associated with knee OA [43], [44], [45]. Furthermore, osteophytes form by endochondral ossification, reactivation of which is thought to play an important role in OA development in adult joints [46], [47], [48]. Polymorphisms associated with OA susceptibility lie close to genes involved in the regulation of endochondral ossification [49]. Interestingly, genetic variation at loci associated with both endochondral ossification and Wnt signalling [50], is greater amongst our HBM population [14]. Therefore, despite lack of temporal data, we consider the relationships we report here, to most likely reflect either a causal pathway between higher BMD and increased risk of ‘bone-forming’ features of OA, or genetic pleiotropy.

It remains theoretically possible that OA features within the DXA field (*e.g.* lumbar osteophytosis) could lead to artefactual elevation of measured BMD, with the potential to induce a spurious HBM-OA association if spine and hand OA are correlated as part of a “generalised OA” phenotype. As discussed, every effort was made within the study design to avoid such misclassification of HBM status; DXA scans were visually inspected for artefactual causes of raised BMD including significant OA, and the L1 vertebra was selected for case definition as L1 Z-scores were not associated with features of OA visible on lumbar DXA [12], [21]. In fact, this approach may have led to some individuals with both HBM and OA being excluded, which if anything would bias our results towards the null. Reassuringly, hip OA has been shown to only minimally influence measured hip BMD [51], and sensitivity analyses limited to HBM cases with hip predominant HBM generated point estimates similar to overall results.

### 4.1 Limitations

Since controls were recruited from within families, they are likely to have greater similarity to HBM cases than unrelated general population controls; hence clustered analyses were performed to account for the lack of statistical independence due to within-family clustering of environmental factors and shared genotypes. Despite this, our reported differences may still underestimate the true magnitude of the HBM phenotype. Considering referral indications for clinical DXA services, our study design most likely accounts for the observed differences between cases and controls in gender, age, post-menopausal status, estrogen replacement and steroid use. However, older age, greater body weight and female gender are all established risk factors for OA [16], [52], and symptomatic hand OA is particularly seen in post-menopausal women [53]. As the HBM cases were older, more likely to be female and post-menopausal, and have a higher BMI than family controls, our results may be explained by residual confounding as regression models may not fully adjust for these marked differences.

Some radiographs for both cases and controls were unavailable; less mobile participants with OA may have been less likely to have attended for X-rays, potentially leading to underestimation of the true OA prevalence within our study population. We lacked data regarding any historic hand joint injuries as well as precise joint specific examination findings, hence our reporting of global hand OA, which meant the potential differences between finger and thumb examination findings could not be determined. Between-centre variations in radiographic protocols may have introduced measurement error; however, in affecting both cases and controls, the expected effect would be to bias our results towards the null. We lack temporal data, so the direction of causality cannot be formally assessed; nevertheless, we assume the onset of genetically determined HBM would predate the onset of OA (a disease of later life) in this population. However, it is theoretically possible that OA features within the DXA field (*e.g.* lumbar osteophytosis) could lead to artefactual elevation of measured BMD, with the potential to induce a spurious association between HBM and OA if spine and hand OA are correlated as part of a “generalised OA” phenotype. As discussed, every effort was made to avoid such misclassification of HBM status through both inspection of DXA images and our case definition; also the fact that the association between HBM and hand OA remained robust when restricted to those HBM cases with high hip BMD is reassuring (Supplementary Table 9), as hip OA is thought to have only a minimal influence on measured hip BMD [51]. A single blinded observer graded all radiographs, which may have led to either over or underestimation of OA prevalence rather than affecting between-group differences; this strategy was chosen as intra-rater repeatability of semi-quantitative OA scoring is substantially superior to that between observers [54]. Ideally all radiographs would have been dual graded, enabling robust calculation of agreement for rarer characteristics.

## 5 Conclusion

In conclusion, our findings support a positive association between HBM and osteophytosis in non-weight-bearing joints of the hand, which is independent of BMI. HBM-associated OA is characterised by osteophytes, consistent with a ‘bone forming’ phenotype, rather than JSN reflecting cartilage loss, and is similar to the phenotype we have previously reported in weight-bearing joints of the knee and hip [9], [10]. However, this ‘bone forming’ hand phenotype is not explained by the lower bone turnover seen in adult HBM cases. It is possible that the same systemic factors, for example genetic architecture, which govern HBM may also increase the risk of osteophytosis.

## Conflicts of interest statement

The authors have no conflicts of interest to declare relevant to this work.

## Funding statement

This work was supported by the Wellcome Trust; CLG was funded through a Clinical Research Training Fellowship [grant ref. 080280/Z/06/Z]; Arthritis Research UK funded SH through a clinical PhD Studentship [grant ref. 19580]. Arthritis Research UK provide ongoing funding for CLG through a Clinician Scientist Fellowship [grant ref. 20000]. Funders had no role in the design, conduct or analysis of this study.

## Figures and Tables

**Table 1:** Semi-quantitative scoring of radiographic features of CMCJ and DIPJ osteoarthritis. Grading of individual radiographic features was performed using the Altman atlas [31]. OP = osteophyte.

OA feature	Categorical grading	Binary variable(s)
Osteophytes	0–3	Any osteophyte (any OP grade ≥ 1), moderate osteophyte (any OP grade ≥ 2)
Joint space narrowing (JSN)	0–3	Any JSN (JSN grade ≥ 1), moderate JSN (JSN grade ≥ 2)
Subchondral sclerosis	0–1	Subchondral sclerosis (grade ≥ 1)
Mal-alignment (DIPJ only)	0–1	Mal-alignment (grade ≥ 1)
Cysts (CMCJ only)	0–1	Cysts (grade ≥ 1)

**Table 2:** Demographics of study population. HBM: High Bone Mass. SD: standard deviation. BMI: body mass index. PA: physical activity. IPAQ: International Physical Activity Questionnaire.

	HBM cases (n = 314)	Controls (n = 183)	p value
	Mean (SD)	Mean (SD)	
Age (years)	61.1 (14.1)	54.3 (16.3)	< 0.001
Height (cm)	167.1 (8.7)	171.1 (10.2)	< 0.001
Weight (kg)	85.6 (16.9)	83.2 (17.4)	0.109
BMI (kg/m^2^)	30.8 (5.8)	28.5 (5.0)	< 0.001
L1 Z-score	3.96 (1.5)	0.56 (1.2)	< 0.001
Total hip Z-score	3.04 (1.2)	0.63 (0.89)	< 0.001
P1NP (μg/L)^a^	36.1 (19.9)	40.7 (22.3)	0.022
Osteocalcin (total) (μg/L)^a^	15.8 (7.9)	18.0 (8.0)	0.003
CTX (μg/L)^a^	0.20 (0.13)	0.24 (0.17)	0.005

	HBM cases (n = 314)	Controls (n = 183)	p value
	n (%)	n (%)	

Female	234 (74.5)	84 (45.9)	< 0.001
Postmenopausal	187 (80.6)	45 (55.6)	< 0.001
Estrogen replacement use (ever)^b^	109 (51.4)	15 (20.8)	< 0.001
Diabetes mellitus	37 (11.8)	12 (6.6)	0.064
Self-reported osteoarthritis	72 (22.9)	28 (15.3)	0.040
Self-reported inflammatory arthritis	20 (6.4)	2 (1.1)	0.013
Steroid use (ever)^c^	82 (26.5)	31 (17.7)	0.030
Previous fracture^d^	120 (38.2)	85 (46.7)	0.065
Current PA (IPAQ) (n = 434)			
Low	45 (14.3)	19 (10.4)	
Moderate	104 (33.1)	53 (29.0)	0.188
High	132 (42.0)	81 (44.3)	
Historical PA score^e^ (n = 431)			
Very low (0–4)	33 (10.5)	17 (9.3)	0.288
Low (5–7)	56 (17.8)	40 (21.9)	
Moderate (8–10)	60 (19.1)	36 (19.7)	
High (11–14)	66 (21.0)	28 (15.3)	
Very high (15–24)	63 (20.1)	32 (17.5)	

a n = 475.

b Post-menopausal estrogen replacement therapy.

c Previous or current, includes eye drops, intra-articular steroid injections and oral steroids for *e.g.* asthma, PMR, ulcerative colitis.

d From any mechanism.

e Constructed using best available evidence, grading PA between 0 (no PA) & 24 (very high PA) [35], [36], [37].

**Table 3:** Unadjusted osteoarthritis variables at the index Distal Interphalangeal Joint (DIPJ) and 1st Carpometacarpal Joint (CMCJ). JSN: joint space narrowing; OR: odds ratio

	HBM cases (n 314)	Controls (n 183)	OR (95% CI)	p value
	n (%)	n (%)		
Symptomatic hand OA^a^				
Clinical history of hand OA	91 (29.0)	31 (16.9)	2.08 (1.29, 3.36)	0.003
Clinical exam suggesting hand OA	89 (28.3)	33 (18.0)	1.89 (1.17, 3.05)	0.009
DIPJ radiographic grading				
Any osteophyte (≥ grade 1)	210 (66.9)	83 (45.4)	2.51 (1.69, 3.73)	< 0.001
Moderate osteophytes (≥ grade 2)	92 (29.3)	26 (14.2)	2.61 (1.58, 4.33)	< 0.001
Any JSN (≥ grade 1)	122 (38.9)	61 (33.3)	1.29 (0.87, 1.91)	0.213
Moderate JSN (≥ grade 2)	22 (7.0)	11 (6.0)	1.20 (0.55, 2.58)	0.649
Subchondral sclerosis	25 (8.0)	6 (3.3)	2.58 (1.02, 6.55)	0.046
Mal-alignment	17 (5.4)	1 (0.5)	11.0 (1.42, 84.9)	0.022
CMCJ radiographic grading				
Any osteophyte (≥ grade 1)	218 (69.4)	92 (50.3)	2.33 (1.56, 3.49)	< 0.001
Moderate osteophytes (≥ grade 2)	94 (29.9)	27 (14.8)	2.55 (1.56, 4.18)	< 0.001
Any JSN (≥ grade 1)	134 (42.7)	73 (39.9)	1.13 (0.77, 1.67)	0.521
Moderate JSN (≥ grade 2)	37 (11.8)	7 (3.8)	3.65 (1.53, 8.71)	0.003
Subchondral sclerosis	38 (12.2)	12 (6.6)	2.05 (1.01, 4.13)	0.045
Cysts	11 (3.5)	1 (0.5)	7.25 (0.90, 58.6)	0.063

a Clinical history and examination findings relate to the same side as was X-rayed for each individual.

**Table 4:** Osteoarthritis variables at the index Distal Interphalangeal Joint (DIPJ) and 1st Carpometacarpal Joint (CMCJ) adjusted for age and sex, with additional adjustment for BMI. JSN: joint space narrowing; OR: odds ratio. Based on 314 HBM cases and 183 controls.

	OR (95% CI)	p value	OR (95% CI)	p value
	Adjusted for age & sex		Adjusted for age & sex & BMI	
Symptomatic hand OA^a^				
Clinical history of hand OA	1.20 (0.72, 1.99)	0.493	1.15 (0.68, 1.94)	0.595
Clinical exam suggesting hand OA	1.03 (0.61, 1.73)	0.908	1.05 (0.62, 1.78)	0.849
DIPJ radiographic grading				
Any osteophyte (≥ grade 1)	1.82 (1.11, 2.97)	0.017	1.72 (1.05, 2.83)	0.032
Moderate osteophytes (≥ grade 2)	1.51 (0.86, 2.65)	0.155	1.47 (0.83, 2.61)	0.183
Any JSN (≥ grade 1)	0.85 (0.54, 1.34)	0.492	0.76 (0.48, 1.21)	0.242
Moderate JSN (≥ grade 2)	0.65 (0.29, 1.46)	0.297	0.65 (0.28, 1.47)	0.301
Subchondral sclerosis	1.22 (0.46, 3.23)	0.687	1.40 (0.52, 3.72)	0.505
Mal-alignment	5.33 (0.67, 42.7)	0.115	4.77 (0.59, 38.5)	0.142
CMCJ radiographic grading				
Any osteophyte (≥ grade 1)	1.89 (1.19, 3.01)	0.007	1.76 (1.10, 2.83)	0.019
Moderate osteophytes (≥ grade 2)	1.85 (1.07, 3.22)	0.028	1.75 (1.00, 3.06)	0.049
Any JSN (≥ grade 1)	0.85 (0.55, 1.31)	0.449	0.77 (0.50, 1.21)	0.256
Moderate JSN (≥ grade 2)	1.87 (0.75, 4.66)	0.181	1.68 (0.67, 4.22)	0.272
Subchondral sclerosis	0.97 (0.46, 2.05)	0.928	1.02 (0.48, 2.18)	0.957
Cysts	3.73 (0.44, 31.5)	0.226	3.71 (0.43, 32.0)	0.233

a Clinical history and examination findings relate to the same side as was X-rayed for each individual.

## Appendix A Supplementary data

Supplementary material Image 1
