# Supplementary material for: High Bone Mass is associated with bone-forming features of osteoarthritis in non-weight bearing joints independent of body mass index
Source: Bone. 2017 Apr;97:306–13. doi: 10.1016/j.bone.2017.01.005 (PMC5378151; doi:10.1016/j.bone.2017.01.005)
Supplement: Supplementary file 1 — Supplementary material [file mmc1.docx]

**Supplementary Tables for ‘High Bone Mass is associated with bone-forming features of osteoarthritis in non-weight bearing joints independent of body mass index’, by Gregson et al 2017.**

**Contents:**

Supplementary table 1: Comparison of individuals do did and did not complete the physical activity questionnaire

Supplementary table 2: Comparison of individuals with and without hand X-rays available

Supplementary table 3: Osteoarthritis variables at the Index Distal Interphalangeal Joint (DIPJ) and 1^st^ Carpometacarpal Joint (CMCJ) adjusted for age, sex and BMI plus menopausal status, diabetes and use of oestrogen replacement and steroids

Supplementary table 4: Osteoarthritis variables at the Index Distal Interphalangeal Joint (DIPJ) and 1^st^ Carpometacarpal Joint (CMCJ) adjusted for age, sex and BMI plus additional adjustment for bone turnover markers (restricted dataset n=475 [304 HBM cases and 171 controls])

Supplementary table 5: Osteoarthritis variables at the Index Distal Interphalangeal Joint (DIPJ) and 1^st^ Carpometacarpal Joint (CMCJ) adjusted for age and BMI, stratified by gender

Supplementary table 6: Osteoarthritis variables at the Index Distal Interphalangeal Joint (DIPJ) and 1^st^ Carpometacarpal Joint (CMCJ), adjusted for age, gender & BMI, excluding individuals with X-rays rated as being of poor quality (n=25)

Supplementary table 7: Osteoarthritis variables at the Index Distal Interphalangeal Joint (DIPJ) and 1^st^ Carpometacarpal Joint (CMCJ), adjusted for age, gender & BMI, excluding individuals reporting an inflammatory arthritis (n=22)

Supplementary table 8: Osteoarthritis variables at the Index Distal Interphalangeal Joint (DIPJ) and 1^st^ Carpometacarpal Joint (CMCJ), adjusted for age, gender & BMI, excluding individuals who reported previous or current steroid use (n=113), or in whom data regarding steroid use were missing (n=12)

Supplementary table 9: Osteoarthritis variables at the Index Distal Interphalangeal Joint (DIPJ) and 1^st^ Carpometacarpal Joint (CMCJ), adjusted for age, gender & BMI, excluding HBM cases with Total Hip Z-score <+3.2 (n=166)

Supplementary figure 1: Standardized BMD (for Total Hip and L1) against age, for HBM cases and controls, stratified by gender

References for Supplementary Data

**Supplementary table 1: Comparison of individuals do did and did not complete the physical activity questionnaire**

HBM: High Bone Mass, SD: Standard Deviation. BMI: Body Mass Index. PAQ: Physical Activity Questionnaire.

|  | **PAQ compete**  **N=434** | **PAQ not completed**  **N=63** | **p value** |
| --- | --- | --- | --- |
|  | Mean (SD) | Mean (SD) |  |
| Height (cm) | 168.4 (9.37) | 169.9 (9.99) | 0.243 |
| Weight (kg) | 84.2 (17.1) | 85.1 (17.6) | 0.705 |
| BMI (kg/m^2^) | 29.7 (5.74) | 29.4 (5.06) | 0.659 |

**Supplementary table 2: Comparison of individuals with and without Hand X-rays available**

HBM: High Bone Mass, CI: Confidence Interval. L1: 1^st^ lumbar vertebra. BMI: Body Mass Index. PA: Physical activity. IPAQ: International Physical Activity Questionnaire.  ^a^ post-menopausal estrogen replacement therapy. ^b^ previous or current, includes eye drops, intra-articular steroid injections and oral steroids for *e.g.* asthma, PMR, ulcerative colitis. ^c^ from any mechanism. ^d^ constructed using best available evidence, grading PA between 0 (no PA) & 24 (very high PA) (1-3)

|  | **Hand X-ray unavailable** | **Hand X-ray available** | **p value** |
| --- | --- | --- | --- |
|  | Mean (95% CI) | Mean (95% CI) |  |
| Age (years) | 61.5 (57.5, 65.5) | 58.6 (57.3, 59.9) | 0.183 |
| Height (cm) | 165.8 (163.3, 168.4) | 168.4 (167.5, 169.3) | 0.057 |
| Weight (kg) | 81.2 (76.4, 86.0) | 84.9 (83.0, 86.8) | 0.145 |
| BMI (kg/m^2^) | 29.7 (28.0, 31.3) | 30.2 (29.5, 30.9) | 0.513 |
| L1 Z-score | 2.79 (2.22, 3.36) | 2.66 (2.47, 2.85) | 0.673 |
| Total Hip Z-score | 2.16 (1.71, 2.60) | 2.09 (1.95, 2.24) | 0.786 |
|  | n (%) | n (%) |  |
| HBM cases | 39 (70.9) | 316 (63.2) | 0.659 |
| Female | 45 (81.8) | 320 (64.0) | 0.032 |
| Postmenopausal | 32 (88.9) | 234 (74.3) | 0.307 |
| Estrogen replacement use (ever)^a^ | 17 (48.6) | 126 (44.1) | 0.969 |
| Diabetes Mellitus | 6 (10.9) | 49 (9.8) | 0.947 |
| Self-reported osteoarthritis | 15 (27.3) | 101 (20.2) | 0.221 |
| Self-reported inflammatory arthritis | 4 (7.3) | 22 (4.4) | 0.598 |
| Steroid use (ever)^b^ | 11 (23.9) | 113 (23.2) | 0.883 |
| Previous fracture^c^ | 19 (37.3) | 205 (41.1) | 0.911 |
| **Self-reported alcohol consumption** | | | |
| None | 12 (21.8) | 108 (21.6) | 0.130 |
| Occasional | 11 (20.0) | 55 (11.0) |  |
| Regular | 18 (32.7) | 247 (49.4) |  |
| Heavy | 3 (5.5) | 78 (15.6) |  |
| **Self-reported smoking status** | | | |
| Never | 32 (58.2) | 238 (47.6) | 0.143 |
| Ex-smoker | 19 (34.5) | 208 (41.6) |  |
| Current | 4 (7.3) | 54 (10.8) |  |
| **Current PA (IPAQ) (n=480)** | | | |
| Low | 12 (21.8) | 64 (12.8) |  |
| Moderate | 15 (27.3) | 159 (31.8) | 0.051 |
| High | 16 (29.1) | 214 (42.8) |  |
| **Historical PA score^d^ (n=477)** | | | |
| Very low (0-4) | 10 (18.2) | 50 (10) | 0.232 |
| Low (5-7) | 8 (14.5) | 97 (19.4) |  |
| Moderate (8-10) | 7 (12.7) | 96 (19.2) |  |
| High(11-14) | 12 (21.8) | 96 (19.2) |  |
| Very high(15-24) | 6 (10.9) | 95 (19) |  |

**Supplementary table 3: Osteoarthritis variables at the Index Distal Interphalangeal Joint (DIPJ) and 1^st^ Carpometacarpal Joint (CMCJ) adjusted for age, sex and BMI plus menopausal status, diabetes, oestrogen replacement, and use of steroids**

JSN: Joint Space Narrowing; OR: Odds Ratio. Based on 314 HBM cases and 183 controls. ^a^ previous or current, includes eye drops, intra-articular steroid injections and oral steroids for *e.g.* asthma, PMR, ulcerative colitis. ^b^ too few observations to run this model.

| (n=497) | OR (95% CI) | p value |
| --- | --- | --- |
|  | Adjusted for age & sex & BMI, menopausal status, diabetes, use of steroids^a^ and of estrogen replacement |  |
| **DIPJ radiographic grading** |  |  |
| Any osteophyte (≥grade 1) | 2.36 (0.95, 5.87) | 0.066 |
| Moderate osteophytes (≥grade 2) | 2.07 (0.78, 5.49) | 0.145 |
| Any JSN (≥grade 1) | 0.91 (0.39, 2.13) | 0.836 |
| Moderate JSN (≥grade 2) | 0.52 (0.14, 1.86) | 0.313 |
| Subchondral sclerosis | 0.97 (0.26, 3.58) | 0.960 |
| Mal-alignment | 4.75 (0.46, 48.7) | 0.190 |
| **CMCJ radiographic grading** |  |  |
| Any osteophyte (≥grade 1) | 1.57 (0.69, 3.53) | 0.281 |
| Moderate osteophytes (≥grade 2) | 2.48 (0.92, 6.71) | 0.073 |
| Any JSN (≥grade 1) | 0.82 (0.38, 1.79) | 0.620 |
| Moderate JSN (≥grade 2) | 1.35 (0.34, 5.35) | 0.667 |
| Subchondral sclerosis | 1.73 (0.53, 5.67) | 0.363 |
| Cysts^b^ |  |  |

**Supplementary table 4: Osteoarthritis variables at the Index Distal Interphalangeal Joint (DIPJ) and 1^st^ Carpometacarpal Joint (CMCJ) adjusted for age, sex and BMI plus additional adjustment for bone turnover markers (restricted dataset n=475 [304 HBM cases and 171 controls])**

JSN: Joint Space Narrowing; OR: Odds Ratio

| **(n=475)** | **OR (95% CI)** | p value | **OR (95% CI)** | p value |
| --- | --- | --- | --- | --- |
|  | Adjusted for age & Sex & BMI |  | Adjusted for age & Sex & BMI, CTX, P1NP, osteocalcin |  |
| **DIPJ radiographic grading** |  |  |  |  |
| Any osteophyte (≥grade 1) | 1.58 (0.93, 2.66) | 0.088 | 1.63 (0.97, 2.75) | 0.067 |
| Moderate osteophytes (≥grade 2) | 1.46 (0.81, 2.63) | 0.208 | 1.52 (0.84, 2.74) | 0.170 |
| Any JSN (≥grade 1) | 0.71 (0.44, 1.16) | 0.172 | 0.75 (0.46, 1.22) | 0.245 |
| Moderate JSN (≥grade 2) | 0.68 (0.29, 1.62) | 0.387 | 0.71 (0.30, 1.69) | 0.439 |
| Subchondral sclerosis | 1.25 (0.46, 3.38) | 0.658 | 1.25 (0.47, 3.37) | 0.655 |
| Mal-alignment | 4.34 (0.53, 35.7) | 0.172 | 4.18 (0.52, 33.8) | 0.180 |
| **CMCJ radiographic grading** |  |  |  |  |
| Any osteophyte (≥grade 1) | 1.85 (1.14, 3.01) | 0.013 | 1.89 (1.15, 3.11) | 0.012 |
| Moderate osteophytes (≥grade 2) | 1.71 (0.96, 3.05) | 0.066 | 1.82 (1.01, 3.27) | 0.045 |
| Any JSN (≥grade 1) | 0.79 (0.49, 1.26) | 0.318 | 0.80 (0.50, 1.27) | 0.339 |
| Moderate JSN (≥grade 2) | 2.00 (0.75, 5.37) | 0.169 | 1.94 (0.72, 5.18) | 0.188 |
| Subchondral sclerosis | 1.08 (0.49, 2.38) | 0.852 | 1.16 (0.52, 2.63) | 0.713 |
| Cysts | 3.61 (0.41, 31.6) | 0.246 | 4.34 (0.46, 40.8) | 0.199 |

**Supplementary table 5: Osteoarthritis variables at the Index Distal Interphalangeal Joint (DIPJ) and 1^st^ Carpometacarpal Joint (CMCJ) adjusted for age and BMI, stratified by gender**

JSN: Joint Space Narrowing; OR: Odds Ratio. No evidence of gender interaction was detected for any variable

|  | **FEMALES (n=318)** | |  |  | **MALES (n=179)** | |  |  |
| --- | --- | --- | --- | --- | --- | --- | --- | --- |
|  | **HBM cases**  **(n=234)** | **Controls**  **(n=84)** | **OR (95% CI)** | p value | **HBM cases**  **(n=80)** | **Controls**  **(n=99)** | **OR (95% CI)** | p value |
|  | **n (%)** | **n (%)** |  |  | **n (%)** | **n (%)** |  |  |
| **DIPJ radiographic grading** |  |  |  |  |  |  |  |  |
| Any osteophyte (≥grade 1) | 152 (65) | 37 (44) | 1.64 (0.77, 3.47) | 0.196 | 58 (72.5) | 46 (46.5) | 1.98 (0.89, 4.39) | 0.093 |
| Moderate osteophytes (≥grade 2) | 71 (30.3) | 14 (16.7) | 1.45 (0.63, 3.34) | 0.376 | 21 (26.3) | 12 (12.1) | 1.52 (0.62, 3.73) | 0.361 |
| Any JSN (≥grade 1) | 90 (38.5) | 27 (32.1) | 0.76 (0.37, 1.55) | 0.451 | 32 (40) | 34 (34.3) | 0.72 (0.35, 1.49) | 0.379 |
| Moderate JSN (≥grade 2) | 17 (7.3) | 8 (9.5) | 0.46 (0.15, 1.36) | 0.159 | 5 (6.3) | 3 (3.0) | 1.12 (0.23, 5.39) | 0.888 |
| Subchondral sclerosis | 22 (9.4) | 5 (6.0) | 1.31 (0.39, 4.36) | 0.663 | 3 (3.8) | 1 (1.0) | 2.46 (0.24, 25.6) | 0.452 |
| Mal-alignment | 15 (6.4) | 1 (1.2) |  |  | 2 (2.5) | 0 (0) |  |  |
| **CMCJ radiographic grading** |  |  |  |  |  |  |  |  |
| Any osteophyte (≥grade 1) | 160 (68.4) | 36 (42.9) | 1.99 (0.99, 4.01) | 0.055 | 58 (72.5) | 56 (56.6) | 1.57 (0.72, 3.45) | 0.259 |
| Moderate osteophytes (≥grade 2) | 71 (30.3) | 9 (10.7) | 2.84 (1.15, 7.01) | 0.024 | 23 (28.8) | 18 (18.2) | 1.14 (0.51, 2.54) | 0.750 |
| Any JSN (≥grade 1) | 100 (42.7) | 28 (33.3) | 0.88 (0.44, 1.76) | 0.717 | 34 (42.5) | 45 (45.5) | 0.57 (0.29, 1.12) | 0.103 |
| Moderate JSN (≥grade 2) | 30 (12.8) | 4 (4.8) | 1.97 (0.55, 7.08) | 0.298 | 7 (8.8) | 3 (3.0) | 1.40 (0.31, 6.44) | 0.663 |
| Subchondral sclerosis | 35 (15.1) | 7 (8.3) | 1.46 (0.52, 4.09) | 0.468 | 3 (3.8) | 5 (5.1) | 0.38 (0.08, 1.88) | 0.238 |
| Cysts | 10 (4.3) | 0 (0) |  |  | 1 (1.3) | 1 (1.0) |  |  |

**Supplementary table 6: Osteoarthritis variables at the Index Distal Interphalangeal Joint (DIPJ) and 1^st^ Carpometacarpal Joint (CMCJ), adjusted for age, gender & BMI, excluding individuals with X-rays rated as being of poor quality (n=25)**

JSN: Joint Space Narrowing; OR: Odds Ratio

| **(n=472)** | **HBM cases**  **(n=295)** | **Controls**  **(n=177)** | **OR (95% CI)** | **p value** |
| --- | --- | --- | --- | --- |
|  | **n (%)** | **n (%)** |  |  |
| **DIPJ radiographic grading** |  |  |  |  |
| Any osteophyte (≥grade 1) | 196 (66.4) | 80 (45.2) | 1.71 (1.03, 2.83) | 0.038 |
| Moderate osteophytes (≥grade 2) | 84 (28.5) | 25 (14.1) | 1.41 (0.79, 2.53) | 0.248 |
| Any JSN (≥grade 1) | 111 (37.6) | 59 (33.3) | 0.72 (0.45, 1.16) | 0.183 |
| Moderate JSN (≥grade 2) | 19 (6.4) | 10 (5.6) | 0.60 (0.25, 1.41) | 0.240 |
| Subchondral sclerosis | 25 (8.5) | 5 (2.8) | 1.72 (0.61, 4.88) | 0.307 |
| Mal-alignment | 16 (5.4) | 1 (0.6) | 4.60 (0.57, 37.4) | 0.153 |
| **CMCJ radiographic grading** |  |  |  |  |
| Any osteophyte (≥grade 1) | 204 (69.2) | 87 (49.2) | 1.85 (1.15, 2.98) | 0.012 |
| Moderate osteophytes (≥grade 2) | 86 (29.2) | 26 (14.7) | 1.72 (0.97, 3.04) | 0.063 |
| Any JSN (≥grade 1) | 120 (40.7) | 69 (39) | 0.75 (0.48, 1.18) | 0.209 |
| Moderate JSN (≥grade 2) | 30 (10.2) | 6 (3.4) | 1.43 (0.54, 3.81) | 0.475 |
| Subchondral sclerosis | 36 (12.3) | 11 (6.2) | 1.10 (0.50, 2.41) | 0.813 |
| Cysts | 9 (3.1) | 1 (0.6) | 3.27 (0.37, 29.0) | 0.287 |

**Supplementary table 7: Osteoarthritis variables at the Index Distal Interphalangeal Joint (DIPJ) and 1^st^ Carpometacarpal Joint (CMCJ), adjusted for age, gender & BMI, excluding individuals reporting an inflammatory arthritis (n=22)**

JSN: Joint Space Narrowing; OR: Odds Ratio

| **(n=475)** | **HBM cases**  **(n=294)** | **Controls**  **(n=181)** | **OR (95% CI)** | **p value** |
| --- | --- | --- | --- | --- |
|  | **n (%)** | **n (%)** |  |  |
| **DIPJ radiographic grading** |  |  |  |  |
| Any osteophyte (≥grade 1) | 196 (66.7) | 83 (45.9) | 1.69 (1.02, 2.79) | 0.040 |
| Moderate osteophytes (≥grade 2) | 83 (28.2) | 26 (14.4) | 1.36 (0.76, 2.43) | 0.295 |
| Any JSN (≥grade 1) | 110 (37.4) | 61 (33.7) | 0.73 (0.46, 1.17) | 0.187 |
| Moderate JSN (≥grade 2) | 19 (6.5) | 11 (6.1) | 0.59 (0.26, 1.38) | 0.226 |
| Subchondral sclerosis | 24 (8.2) | 6 (3.3) | 1.46 (0.55, 3.88) | 0.447 |
| Mal-alignment | 13 (4.4) | 1 (0.6) | 3.85 (0.47, 31.4) | 0.207 |
| **CMCJ radiographic grading** |  |  |  |  |
| Any osteophyte (≥grade 1) | 206 (70.1) | 92 (50.8) | 1.89 (1.16, 3.07) | 0.010 |
| Moderate osteophytes (≥grade 2) | 89 (30.3) | 27 (14.9) | 1.83 (1.04, 3.22) | 0.035 |
| Any JSN (≥grade 1) | 125 (42.5) | 72 (39.8) | 0.77 (0.49, 1.21) | 0.263 |
| Moderate JSN (≥grade 2) | 35 (11.9) | 7 (3.9) | 1.70 (0.67, 4.32) | 0.260 |
| Subchondral sclerosis | 33 (11.3) | 12 (6.6) | 0.94 (0.43, 2.03) | 0.870 |
| Cysts | 10 (3.4) | 1 (0.6) | 3.75 (0.43, 32.4) | 0.230 |

**Supplementary table 8: Osteoarthritis variables at the Index Distal Interphalangeal Joint (DIPJ) and 1^st^ Carpometacarpal Joint (CMCJ), adjusted for age, gender & BMI, excluding individuals who reported previous or current steroid use (n=113), or in whom data regarding steroid use were missing (n=12)**

JSN: Joint Space Narrowing; OR: Odds Ratio

| **(n=372)** | **HBM cases**  **(n=228)** | **Controls**  **(n=144)** | **OR (95% CI)** | **p value** |
| --- | --- | --- | --- | --- |
|  | **n (%)** | **n (%)** |  |  |
| **DIPJ radiographic grading** |  |  |  |  |
| Any osteophyte (≥grade 1) | 148 (64.9) | 63 (43.8) | 1.88 (1.05, 3.37) | 0.033 |
| Moderate osteophytes (≥grade 2) | 69 (30.3) | 20 (13.9) | 1.51 (0.77, 2.95) | 0.229 |
| Any JSN (≥grade 1) | 83 (36.4) | 44 (30.6) | 0.84 (0.48, 1.47) | 0.542 |
| Moderate JSN (≥grade 2) | 17 (7.5) | 8 (5.6) | 0.77 (0.30, 2.02) | 0.601 |
| Subchondral sclerosis | 19 (8.3) | 5 (3.5) | 1.25 (0.42, 3.74) | 0.692 |
| Mal-alignment | 12 (5.3) | 1 (0.7) | 4.36 (0.52, 36.9) | 0.177 |
| **CMCJ radiographic grading** |  |  |  |  |
| Any osteophyte (≥grade 1) | 153 (67.1) | 70 (48.6) | 1.88 (1.11, 3.20) | 0.019 |
| Moderate osteophytes (≥grade 2) | 65 (28.5) | 21 (14.6) | 1.62 (0.84, 3.11) | 0.151 |
| Any JSN (≥grade 1) | 97 (42.5) | 57 (39.6) | 0.77 (0.46, 1.29) | 0.323 |
| Moderate JSN (≥grade 2) | 28 (12.3) | 7 (4.9) | 1.33 (0.50, 3.52) | 0.572 |
| Subchondral sclerosis | 25 (11) | 10 (6.9) | 0.74 (0.31, 1.77) | 0.492 |
| Cysts | 9 (4) | 1 (0.7) | 3.23 (0.36, 28.9) | 0.295 |

**Supplementary table 9: Osteoarthritis variables at the Index Distal Interphalangeal Joint (DIPJ) and 1^st^ Carpometacarpal Joint (CMCJ), adjusted for age, gender & BMI, excluding HBM cases with Total Hip Z-score <+3.2 (n=166)**

JSN: Joint Space Narrowing; OR: Odds Ratio

|  | **HBM cases**  **(n=148)** | **Controls**  **(n=183)** | **OR (95% CI)** | **p value** |
| --- | --- | --- | --- | --- |
|  | **n (%)** | **n (%)** |  |  |
| **DIPJ radiographic grading** |  |  |  |  |
| Any osteophyte (≥grade 1) | 98 (66.2) | 83 (45.4) | 1.47 (0.80, 2.72) | 0.217 |
| Moderate osteophytes (≥grade 2) | 48 (32.4) | 26 (14.2) | 1.71 (0.87, 3.34) | 0.119 |
| Any JSN (≥grade 1) | 62 (41.9) | 61 (33.3) | 0.89 (0.51, 1.54) | 0.666 |
| Moderate JSN (≥grade 2) | 10 (6.8) | 11 (6) | 0.60 (0.22, 1.63) | 0.314 |
| Subchondral sclerosis | 15 (10.1) | 6 (3.3) | 1.87 (0.64, 5.45) | 0.253 |
| Mal-alignment | 7 (4.7) | 1 (0.5) | 2.29 (0.26, 20.5) | 0.458 |
| **CMCJ radiographic grading** |  |  |  |  |
| Any osteophyte (≥grade 1) | 109 (73.6) | 92 (50.3) | 2.03 (1.12, 3.69) | 0.020 |
| Moderate osteophytes (≥grade 2) | 45 (30.4) | 27 (14.8) | 1.61 (0.84, 3.11) | 0.151 |
| Any JSN (≥grade 1) | 75 (50.7) | 73 (39.9) | 0.95 (0.54, 1.65) | 0.849 |
| Moderate JSN (≥grade 2) | 19 (12.8) | 7 (3.8) | 1.41 (0.51, 3.94) | 0.511 |
| Subchondral sclerosis | 26 (17.7) | 12 (6.6) | 1.86 (0.79, 4.38) | 0.153 |
| Cysts | 6 (4.1) | 1 (0.5) | 4.14 (0.40, 42.9) | 0.234 |

**Supplementary figure 1: Standardized BMD (for Total Hip and L1) against age, for HBM cases and controls, stratified by gender**

|  | **Females** | **Males** |
| --- | --- | --- |
| **Total Hip sBMD** | **** | **** |
| **L1 sBMD** | **** | **** |

Red dots=HBM cases. Blue crosses=Controls

**References for Supplementary Material**

1. Kriska AM, Sandler RB, Cauley JA, LaPorte RE, Hom DL, Pambianco G. The assessment of historical physical activity and its relation to adult bone parameters. Am J Epidemiol. 1988;127(5):1053-63.

2. Suleiman S, Nelson M. Validation in London of a physical activity questionnaire for use in a study of postmenopausal osteopaenia. J Epidemiol Community Health. 1997;51(4):365-72.

3. Chasan-Taber L, Erickson JB, McBride JW, Nasca PC, Chasan-Taber S, Freedson PS. Reproducibility of a self-administered lifetime physical activity questionnaire among female college alumnae. Am J Epidemiol. 2002;155(3):282-9.
